# Post-operative oral chemoprophylaxis in patients undergoing hip arthroscopy mitigates VTE risk with a low side-effect profile

**DOI:** 10.1093/jhps/hnaa063

**Published:** 2020-12-22

**Authors:** Wesley A M Verhoogt, Jurek R T Pietrzak, Olufemi R Ayeni, Josip N Cakic

**Affiliations:** 1 Department of Orthopaedic Surgery, University of the Witwatersrand, Charlotte Maxeke Johannesburg Academic Hospital, Parktown, Johannesburg, 2193, South Africa; 2 Division of Orthopaedic Surgery, McMaster Children's Hospital,1200 Main Street West, Hamilton, ON, L8N 3Z5, Canada; 3 Centre for Sports Medicine and Orthopedics, Fourways Life Hospital, Cedar Ave West &, Cedar Rd, Fourways, Johannesburg, 2055, South Africa

## Abstract

Hip arthroscopy (HA) has increased exponentially over the last decade. A recent systematic review found that the risk of venous thromboembolism (VTE) is 2%. This was higher than previous reports which may have underestimated the true incidence of VTE in HA. Thus, protocols to mediate VTE may be more necessary than previously thought. The aim of this article is to present a VTE prevention protocol and evaluate its subsequent efficacy. This is a prospective study of 880 consecutive HA cases. All patients were treated according to a predetermined VTE protocol which classified patients as high (≥1 risk factors) or low (no risk factors) risk for post-operative VTE. In high-risk patients, the protocol followed that of low-risk patients but additionally included rivaroxaban for 2 weeks post-operatively. The incidence of VTE was recorded and analysed in this study. A total of 880 HA cases at an average age of 35.4 years were evaluated, with 76.6% (n = 674) undergoing labral repair and concomitant cam and/or pincer resection, 17.2% (n = 151) of cases for isolated labral tear repaired, and 6.1% (n = 55) classified as other. The overall incidence of VTE was 0.45%. The incidence of VTE was 1.2% and 0.16% in high- and low-risk groups, respectively. Oral VTE prophylaxis was not associated with post-operative complications. This study demonstrated a lower rate of VTE in both risk groups. It highlights the value of a predetermined risk-adjusted protocol to VTE prophylaxis. Rivaroxaban prophylaxis is safe and efficacious in HA with a low associated morbidity.

## INTRODUCTION

Venous thromboembolism (VTE) is a well-recognized and preventable complication of orthopaedic surgery, often associated with significant morbidity and financial burden [1, 2]. VTE comprises a spectrum ranging from asymptomatic deep vein thromboses (DVT) to life-threatening pulmonary emboli [3]. Post-thrombotic syndrome is a common significant sequelae of VTE occurring in 20–50% of cases and is associated with significant long-term chronic pain, hyperpigmentation, oedema, pruritis, paraesthesia and/or ulceration [1, 2, 4]. VTE may further burden patients and the health care system by resulting in VTE recurrence, chronic thrombotic pulmonary hypertension and complications associated with the treatment of VTE [1, 4]. A conservative estimate in 2016 suggested that VTE cost the US healthcare system $7–10 billion, with 375 000–425 000 cases each year in total [5].

Orthopaedic surgery is a pertinent risk factor for VTE, with incidence estimates of 40–60% for all major orthopaedic procedures when no form of prophylaxis is used [6, 7]. Comparatively, the incidence is 10–30% in medical and general surgery patients [3, 7]. Considering this, VTE prophylaxis guidelines have been developed for certain specific orthopaedic procedures in order to tailor prophylaxis for high-risk and low-risk patients [8]. No formal VTE prophylaxis guidelines currently exist for hip arthroscopy (HA) [8, 9].

The demand for HA has increased exponentially, with increased incidence rates of 727% between 2002 and 2013 and a predicted increases of 1338% by 2023 [10]. HA has become a common technique used in the treatment of hip pathology with good clinical outcomes, low rates of complications and shorter rehabilitation periods in comparison to open surgery [11–13]. Overall complication rates of HA have been reported as 3.3%, with VTE being an important recognised complication [14]. Significant differences in VTE rates following HA have been published. In a 2017 systematic review of 36 761 cases, Nakano *et al*.’s [14] systematic review reported the rate of PE and DVT at 0.01% and 0.09%, respectively. In contrast, in 2016, Fukushima *et al*. described that VTE complicated 6.9% of HA [11, 14]. Despite the low general complication rate, the increased number of HA procedures being performed has come in tandem with a rapid evolution of the surgical instrumentation and an expansion of potential surgical indications. No accepted formal guidelines currently exist for VTE prophylaxis in HA, with the NICE guidelines or EFORT Open Reviews making no recommendations [7, 15].

A paucity of literature regarding VTE complicating HA exists. This study subsequently aimed to establish the incidence of VTE following HA through a prospective trial which enrolled all consecutive HA performed by a single surgeon at a high-volume centre from 2012 to 2018 using a novel, standardized VTE prophylaxis protocol.

## ETHICAL APPROVAL

This research has been approved by The South African Medical Association Research Ethics Committee—Hip Arthroscopy Registry Rev: II 25-08-10.

## MATERIALS AND METHODS

We conducted a prospective study outlined by a predetermined protocol which comprised 880 HA performed between 2012 and 2018 by a single, high-volume orthopaedic surgeon in 646 patients. In patients where bilateral staged HA were performed, each HA was counted as an index primary HA. A manual review of all patients’ standardized clinical data collection forms was performed by two reviewers (WAMV and JRTP). The following demographic data were captured for all patients: age (years), weight (kg), height (m), body mass index (kg/m^2^), ASA grade, medical co-morbidities (including history of previous VTE, diabetes, hypertension, epilepsy, asthma and heart disease), and current chronic medication.

All patients included in this study were followed up for a minimum of 1-year post-operatively, with a 100% follow-up rate. All patients were seen by the senior surgeon at routine clinical follow-up at 3 weeks, 6 weeks, 3 months, 6 months, 9 months and 1-year post-operatively. Both medical and surgical complications were recorded and tabulated. The Clavien–Dindo Classification System was also used to describe the post-operative complications [16].

All patients were clinically evaluated by the senior surgeon at least 6 weeks prior to surgery. An magnetic resonance imaging arthrogram of the index hip was mandatory within 30 days of the case. Patients were stratified into high-risk and low-risk patients for VTE based on their pre-operative work-up. The risk stratification system was based on the Scottish Intercollegiate Guideline Network risk assessment tool [17]. All patients with one or more risk factors for VTE were treated as high-risk cases. Correspondingly, the patients with no risk factors were treated as low-risk cases. Risk factors screened for can be found in the Supplementary Appendix S1 [18]. This determined whether the patients received pharmacological VTE prophylaxis or not. All low-risk patients (no risk factors) were treated with a standardized protocol, as outlined in Table I, which comprised a preoperative work-up, fascia illiaca block, limited traction time and early post-operative mobilization. All high-risk patients (one or more risk factors) received 10 mg of rivaroxaban orally post-operatively for 2 weeks in addition to following the standard VTE prevention protocol which was followed by all low-risk patients.

**Table I.** Venous thromboembolism preventative measures

Pre-operatively	Extensive preoperative history and workup—looking specifically for risk factors of VTEs
	A physician work-up for any patients with a known co-morbidity for optimization prior to surgery
Operatively	Fascia Illica block prior to the procedure—allowing for effective analgesia post operatively to encourage post-operative mobility within 24 h
	A maximum traction time of 2 h
Post-operatively	Early mobilization with physiotherapy assistance within 24 h after surgery
	Two weeks of Rivaroxaban 10 mg orally daily post-operatively in patients with one or more VTE risk factors

Preoperative diagnosis was confirmed intraoperatively and correlated with history, clinical findings and radiographic evidence. Patients were classified according to diagnoses as listed in Table II.

**Table II.** **Demographics** and spectrum of pathology

Demographics	Number (% of total)
Total	880 (100%)
Primary HA	780 (88.6%)
Revision HA	100 (11.4%)
Age	35.14 (13–65)
Gender (M/F)	357 (40.6%)/523 (59.4%)
BMI	24.4 (15.2–39.4)
**Diagnosis**	
Cam and pincer	242 (27.5%)
Cam and labrum	218 (24.74%)
Cam only	147 (16.7%)
Pincer and labrum	67 (7.6%)
Labrum only	151 (17.2%)
Other	55(6.1%)
Surgical time (min)	95 ± 8.3
Traction time (min)	71 ± 4.7

All anaesthetics were given by one of two senior anaesthesiologists. The anaesthetic was performed as a general anaesthetic and was complimented by a fascia illiaca block, using a 50 ml 0.25% Macaine with adrenaline. All patients were positioned in the supine position on a traction table with a post to aid joint distraction. Total traction time was <120 min in all cases.

Post-operatively, all patients were given 12 h of ice pack dressing and were mobilised by a physiotherapist within 24 h post-operatively. Rehabilitation was initiated within 24 h starting in hospital, followed by a home exercise program, and out-patient physiotherapy program 5 days post-operatively.

All patients were thoroughly educated about the risks and, subsequently, the signs and symptoms of VTE. Patients were instructed to follow-up urgently with the surgeon should these symptoms arise. At all standardized follow-up visits (at 3 weeks, 6 weeks, 3 months, 6 months and 1 year post-operatively) signs, such as calf pain, increasing calf circumference, the presence of Homan’s sign, shortness of breath, blood-streaked sputum, and/or chest pain, prompted further investigation immediately [19]. Any suspected symptomatic VTE following HA was confirmed by a combination of clinical examination, serological investigations, duplex doppler and, if indicated, contrast-enhanced computed tomography of the pulmonary vasculature. All confirmed VTE cases were referred to a physician for VTE management. Patients with both symptomatic features and corresponding confirmatory tests were considered as a positive VTE in this study.

## RESULTS

A total of 880 HA were performed, which comprised 646 primary unilateral HA, 134 bilateral procedures completed at separate sittings and 100 revision hip arthroscopies (25 revision cases where the initial arthroscopy was performed elsewhere) between January 2012 and December 2018. The average age was 35.1 years with 40.6% and 59.4% split between males and females, respectively. The follow-up rate for the study was 100%. The average body mass index was 24.4. A spectrum of pathology was seen (Table II) with 76.6% of the cases being femoroacetabular impingement (FAI) related and 23.3% being non-FAI related. The total operative time was 95 ± 8.3 min, while the traction time was 71 ± 4.7 min. Positive VTE risk factors found are listed in Table III. The most common risk factor was the combined oral contraceptive (COC) pill in 17.7%, followed by patients with a body mass index (BMI) >30 at 6.7%. Patients with a previous history of VTE and a family history of VTE were 2.5% and 2.4%, respectively. Hormone replacement therapy was observed in 1.7% and diabetes mellitus in 1.25%. The other risk factors found within the population were <1%.

**Table III.** Risk factors for VTE

Risk factors	Primary HA	Revision HA	Total
Combined oral contraceptives	122	24	156 (17.7%)
Obesity (BMI >30)	50	9	59 (6.7%)
Previous VTE	16	6	22 (2.5%)
Family history	18	3	21 (2.4%)
Hormone-replacement therapy	15	0	15 (1.7%)
Diabetes mellitus	11	0	11 (1.3%)
Known cardiac pathology	4	0	4 (0.4%)
Known malignancy	6	0	6 (0.7%)
Steroids: anabolic	2	0	2 (0.2%)
Age (>65)	1	0	1 (0.1%)

Table IV summarizes the complications found while the protocol was instituted and classifies the complications according to the Clavien–Dindo Classification System [16]. We noted a total VTE incidence rate of 0.45% (confidence interval, 0.010–0.899). The DVT incidence rate was 0.34%, and the PE incidence rate was 0.11%. Three VTE cases were noted in the high-risk patient group and one in the low-risk patient group. This equated to VTE risks of 1.2% and 0.16% in the high- and low-risk groups, respectively. No cases of significant post-operative bleeding occurred. No other major complications were noted with non-VTE related rates consistent with the findings stated in the literature [14].

**Table VI.** Complications

Complications	Cases (% of total)	Complications on VTE prophylaxis (high VTE risk group)	Complications without VTE prophylaxis (low VTE risk group)
Deep vein thrombosis	3 (0.3%)	2	1
Pulmonary embolism	1 (0.1%)	1	0
Myocardial infarction	0 (0%)	0	0
Constant irritation not otherwise specified	3 (0.3%)	0	3
Neuropraxia/sciatica	4 (0.5%)	0	4
Scar-related complications	2 (0.2%)	1	1
Stiffness	5 (0.6%)	2	3
Sepsis (local and systemic)	3 (0.3%)	1	2
Post-operative bleeding	0 (0%)	0	0
Ankle pain	1 (0.1%)	1	0
Clivien–Dindo classification			
I	15	5	10
II	6	3	3
IIIa	0	0	0
IIIb	0	0	0
IVa	1	1	0
IVb	0	0	0

**Table V.** VTE cases

VTE cases	Age/gender	Body mass index	Pathology	No of risk factors	Risk factors for VTE	VTE prophylaxis	Complication (and time to diagnosis)	Previous hip arthroscopy (complications)
Case 1	35/female	27	Cam and pincer	0	Nil	No (low-risk group—no chemo-prophylaxis)	DVT (3 weeks post-operatively)	Nil
Case 2	37/female	23	Labral tear	1	Previous DVT	Yes (Rivaroxaban for 2 weeks)	DVT (3 weeks post-operatively)	Nil
Case 3	39/female	23	Cam only	2	Type 2 diabetes mellitus	No prophylaxis, (PE diagnosed and treated prior to prophylaxis given)	Pulmonary embolism (immediately post-operatively)	12 months prior (no complications)
					Cardiac pathology			
Case 4	41/female	26	Scarring, Cam and pincer	3	Family history of DVT	Yes (Rivaroxaban for 2 weeks)	DVT (3 weeks post-operatively)	15 Months prior (no complications)
					Previous DVT			
					Combined oral contraceptive			

**Table VI.** The rate of VTE based on preoperative risk factors

Preoperative risk factors (risk category)	Total cases	VTE rate based on the number of risk factors (number of cases)	VTE rate based on risk category	Total VTE rate
0 (low risk)	629	0.16% (1)	0.16%	0.45%, CI:0.010–0.899
1 (high risk)	225	0.44% (1)	1.2%	
2 (high risk)	20	5% (1)	1.2%	
3 (high risk)	6	16.7% (1)	1.2%	
4 (high risk)	0	0 (0)	1.2%	

The cases of VTE within the group are displayed in Table V. Four cases of VTE were found, each with varying risk factors. Two cases of VTE developed despite rivaroxaban prophylaxis. Two revision cases that developed VTE (1 DVT and 1 PE) had an uneventful primary HA performed more than 12 months previously where the same prophylaxis protocol was used.

Table VI shows the VTE rate based on the number of risk factors identified. This study observed an increased rate of VTE with increasing risk factors for VTE. One case of VTE was observed out of 629 patients with no risk factors (0.16%) in comparison to 1 in 20 (5%) and 1 in 6 (16.7%) for patients with 2 and 3 risk factors, respectively.

## DISCUSSION

The overall rate of VTE in 880 elective HA was 0.45% (0.16% in low-risk patients and 1.2% in high-risk patients). Moreover, the incidence of DVT and PE was 0.3% and 0.1%, respectively. All surgical procedures were performed by a single, high-volume surgeon in a single institution. VTE is a common peri-operative complication and poses a greater risk in surgical than medical patients [7]. VTE remains a significant, but preventable, cause of both morbidity and mortality after orthopaedic surgery [15, 20]. The problem with VTE in major or more-invasive orthopaedic procedures are well-documented [7, 15, 21, 22]. However, issues regarding the incidence and prevention of DVT and PE after HA remain a diagnostic and therapeutic challenge palpably less clear due to a lack of complete definitive investigations on this topic [9].

The incidence of VTE in HA is reported in the literature as 0–6.94% [9, 23, 24]. Haldane *et al*. [9] published a systematic review that included 21 studies which included studies using clinical and ultrasound surveillance, and they stressed that information regarding VTE and HA remains limited. The incidence of DVT was reported to be 2% in patients where VTE prophylaxis was used and 2.3% in the group of patients who did not take VTE prophylaxis.

Several studies in the literature have described the incidence of VTE in HA in patients who did not receive any prophylaxis, neither mechanical nor pharmacological. Salvo *et al*. [25] reported an incidence of clinically symptomatic DVTs of 3.7%. Similarly, in a prospective analysis of 115 elective HAs which excluded high-risk patients, Mohtadi *et al*. [24] used clinical examination and duplex Doppler ultrasonography to report a DVT rate of 4.3%. These authors stressed the need for routine screening and/or VTE prophylaxis of all patients undergoing HA. Alaia *et al*. [26] reported that the incidence of VTE was 1.4% in a four surgeon series of 139 patients. High-risk patients were excluded and post-operative ultrasonography found no asymptomatic DVTs.

It is controversial whether patients undergoing HA should receive VTE prophylaxis. Bolia *et al*. [27] performed a meta-analysis of 4572 cases from 38 studies and evaluated the incidence of VTE in low-risk patients undergoing HA for FAI. The incidence of DVT and PE was 1.18% and 0.59%, respectively. Once a correction for publication bias was made, the proportion for DVT was actually 2.02%. No mention in this study was made of prophylaxis. However, a multi-centre study reported an overall post-operative complication rate of 8.35% with a clinically determined incidence of VTE of 0.2% in 1502 patients [28]. DVT prophylaxis consisted of compression stockings and early mobilization for most patients. However, high-risk patients were identified as those with a previous history of VTE, clotting disorder or need to fly within 3 weeks of surgery and treated with aspirin prophylaxis of 650 mg daily for 2 weeks [28]. Similarly, our study aimed to identify patients at risk for VTE and treat appropriately with chemoprophylaxis and compare the incidence of VTE thereafter.

A number of risk factors for VTE have been reported. The Scottish Intercollegiate Guidelines Network (SIGN) [18] listed the following risk factors for VTE: age, obesity, varicose veins, a family history of VTE, thrombophilia, COC, hormone replacement therapy (HRT), anti-oestrogens, central venous catheters and immobility [18]. Known risk factors for VTE in patients undergoing other arthroscopic procedures include COC use, smoking, obesity and increasing age [29]. Haldane *et al*. [9] reported a greater likelihood of DVT after HA in patients older than 45 years old, diabetics and those with chronic pulmonary disease. Khazi *et al*. [29] retrospectively reported that the incidence of post-operative VTE in 9477 HAs from a large national database was 0.77% and 1.14% at 30 and 90 days. The study reported that smoking, obesity and diabetes were significant risk factors for VTE [29]. Both gender and COC did not have any noteworthy bearing on VTE, however [29].

Overall, VTE prophylaxis is reported as 13–70% and considerable variation in protocols exist between surgical procedures, institutions and even countries [7, 30, 31]. To the authors’ knowledge, this article is the first to document selective VTE prophylaxis for HA using rivaroxaban. Despite this study, a paucity of literature exists regarding the optimal role of these modalities and best interventional strategies for VTE prophylaxis in HA.

Much debate exists over the ideal chemical anti-coagulant for VTE prophylaxis. A good balance between efficacy and a low-side effect profile is essential. Rivaroxaban was chosen as it allowed for a fixed, oral, once-daily dose with no subsequent monitoring required [7]. Rivaroxaban acts as a direct inhibitor of activated factor Xa [7]. During protocol development, the choice of rivaroxaban over aspirin was made based on numerous guidelines for VTE prevention in orthopaedic surgery. The SIGN guidelines published in 2010 stated that other drugs are more effective for the prevention of VTE, as such aspirin was not recommended for VTE prophylaxis in orthopaedic patients [18]. As this study progressed, the use of aspirin in VTE prophylaxis has gained traction, with the AAOS and the ACCP in 2016 recommending aspirin for arthroplasty VTE prophylaxis, however a metanalysis by An *et al*. [32] in 2016 stated that the evidence available is of limited quality and still remains controversial regarding dosage and duration of therapy. Aspirin for VTE prophylaxis has also been controversial and was published after the protocol for this study was established. Collins *et al*. [33] in 2015 prescribed a daily dose of aspirin 325 mg for 2 weeks after HA and reported a 6.9% rate of VTE. A lower rate of DVT and PE, 0.5% and, 0.2% respectively, was described by Domb *et al*. [34] after administering aspirin 325 mg twice daily after HA for 2 weeks. In the absence of accurately defined guidelines for aspirin use and with rivaroxaban approved for VTE prophylaxis in arthroplasty, all high-risk patients were treated with 2 weeks of oral rivaroxaban.

A limitation of the use of rivaroxaban is the cost difference between aspirin and rivaroxaban, rivaroxaban being significantly more expensive. Wells *et al*. [35] published a paper in CHEST, in 2018, which found that rivaroxaban per patient per month (PPPM) was $24 higher in comparison to aspirin; however, aspirin had three times higher clinical event costs per patient per month in comparison to rivaroxaban in $123 versus $381, respectively.

In our study, there were no cases of wound drainage or bleeding post-operatively and subsequently no related 30-, 60- or 90-day readmissions.

The description of post-operative DVTs is poorly defined [36, 37]. Mostly, they arise within the first week after surgery but may occur up to 3 months post-operatively [36, 38]. The risk of VTE may, therefore, persist beyond the first month after index surgery [39, 40]. Khazi *et al*. [29] reported that the incidence of VTE almost doubled from the first 30 days to 90 days post-operatively. Despite research generally reporting that the causal relationship between surgery and DVT is tenuous after as little as 4 weeks, our study continued to clinically evaluate patients for evidence of VTE, at set clinical time points, up to 1 year post-operatively.

### Limitations

A weakness of this study is that it only evaluated the incidence of symptomatic, clinically relevant VTEs. No post-operative imaging was performed for asymptomatic VTE as no guideline body has ever recommended it. In a retrospective analysis of 72 HAs managed peri-operatively without VTE prophylaxis, Fukushima *et al*. [11] reported five symptomatic, post-operative DVTs (6.94%). No further asymptomatic DVTs were described despite ultrasound surveillance and D-dimer level comparisons pre- and post-operatively [11]. Studies for DVT diagnosis demonstrate that a negative ultrasound result does not completely rule out the possibility of an asymptomatic DVT [41]. Symptomatic DVTs are more common [3, 36] and their clinical outcome far more important than those of asymptomatic DVTs [22, 42, 43]. Asymptomatic DVTs have a good prognosis irrespective of anticoagulation therapy [22, 42]. Asymptomatic DVTs also infrequently cause PEs. Alternatively, 40–50% of untreated symptomatic DVTs will progress to pulmonary emboli [44].

The ninth edition of the ACCP guidelines do not recommend routine peri-operative screening for asymptomatic patients in orthopaedic surgery, as no clinical benefit has been reported [22, 42, 43, 45]. In arthroplasty cases, the ACCP states that routine doppler ultrasound following joint replacement prior to discharge is no longer recommended and it does not make any recommendation regarding routine follow-up screening imaging for asymptomatic surveillance [22]. The authors, however, do concede that asymptomatic DVTs may have existed and were not included in this analysis, possibly underestimating the proportion of VTE. Future research could establish the risk of asymptomatic VTE diagnosed on ultrasound, comparing rivaroxaban versus other agents as a methods of anticoagulation following HA.

The authors acknowledge an inherent surveillance bias, linked in part to the controversy regarding optimal DVT screening. This study aimed to limit this bias by coupling comprehensive patient education with routine, regular single surgeon clinical follow-up and examination of all cases at pre-determined points over 1 year. This method of VTE surveillance has been used and accepted in prior studies tracking VTE complicating both HA and other major orthopaedic procedures [24, 46–48]. Previous research has reported that due to the dire and lethal outcome of complications of VTE, an incidence rate of 1–2% cannot be ignored in clinical practice [27]. Our article, however, supports the knowledge that VTE after HA is low, and certainly comparably less than in other common arthroscopic procedures [34, 49]. We suggest that the incidence of VTE can be decreased safely and effectively using a risk-adjusted approach, by identifying patients with risk factors for VTE and subsequently treating high-risk patients for 2 weeks with an oral anticoagulant. Further clinical trials should be established to investigate these proposed preventative measures to improve current VTE prophylaxis guidelines in HA, with the recognition that each patient needs to be evaluated on a case-by-case basis.

## CONCLUSION

This study demonstrates the value of a formalised risk-adjusted VTE protocol. A reduced rate of VTE in both low-risk (no prophylaxis) and high-risk (chemoprophylaxis used) individuals was shown with a VTE incidence of 0.16% and 1.2%, respectively. Particular attention to females on the COC and patients with more than one risk factor for VTE needs to be considered. Oral VTE chemoprophylaxis (rivaroxaban) for 2 weeks in high-risk patients (with one or more risk factors for VTE) is safe and effective for VTE prophylaxis with a low side-effect profile. 

## SUPPLEMENTARY DATA

Supplementary data are available at *Journal of Hip Preservation Surgery* online.

## Supplementary Material

hnaa063_Supplementary_Data

## References

[1] Ruppert A , Lees M, Steinle T. Clinical burden of venous thromboembolism. Curr Med Res Opin 2010; 26: 2465–73. 20825270 10.1185/03007995.2010.516090

[2] Ruppert A , Steinle T, Lees M. Economic burden of venous thromboembolism: a systematic review. J Med Econ 2011; 14: 65–74. 21222564 10.3111/13696998.2010.546465

[3] Geerts WH , Bergqvist D, Pineo GF et al Prevention of venous thromboembolism. Chest 2008; 133: 381S–453S. 18574271 10.1378/chest.08-0656

[4] Rathbun S. The surgeon general’s call to action to prevent deep vein thrombosis and pulmonary embolism. Circulation 2009; 119: e480–82. 19380627 10.1161/CIRCULATIONAHA.108.841403

[5] Grosse SD , Nelson RE, Nyarko KA et al The economic burden of incident venous thromboembolism in the United States: a review of estimated attributable healthcare costs. Thromb Res 2016; 137: 3–10. 26654719 10.1016/j.thromres.2015.11.033 PMC4706477

[6] Scheres LJJ , Lijfering WM, Cannegieter SC. Current and future burden of venous thrombosis: not simply predictable. Res Pract Thromb Haemost 2018; 2: 199–208. 30046722 10.1002/rth2.12101 PMC6055567

[7] Flevas DA , Megaloikonomos PD, Dimopoulos L et al Thromboembolism prophylaxis in orthopaedics: an update. EFORT Open Rev 2018; 3: 136–48. 29780621 10.1302/2058-5241.3.170018 PMC5941651

[8] Pai M , Douketis JD. Prevention of venous thromboembolism in adult orthopedic surgical patients. UpToDate 2019. Available at: https://www.uptodate.com/contents/prevention-of-venous-thromboembolism-in-adult-orthopedic-surgical-patients. Accessed: 10 January 2020.

[9] Haldane CE , Ekhtiari S, de Sa D et al Venous thromboembolism events after hip arthroscopy: a systematic review. Arthroscopy 2018; 34: 321–30.e1. 28969946 10.1016/j.arthro.2017.07.006

[10] Palmer AJR , Malak TT, Broomfield J et al Past and projected temporal trends in arthroscopic hip surgery in England between 2002 and 2013. BMJ Open Sport Exerc Med 2016; 2: e000082. 10.1136/bmjsem-2015-000082 PMC5117047 27900161

[11] Fukushima K , Takahira N, Uchiyama K et al The incidence of deep vein thrombosis (DVT) during hip arthroscopic surgery. Arch Orthop Trauma Surg 2016; 136:1431–5. 27402212 10.1007/s00402-016-2508-7

[12] Bedi A , Kelly BT, Khanduja V. Arthroscopic hip preservation surgery: current concepts and perspective. Bone Joint J 2013; 95-B: 10–9. 23307667 10.1302/0301-620X.95B1.29608

[13] Griffiths EJ , Khanduja V. Hip arthroscopy: evolution, currentpractice and future developments. Int Orthop 2012; 36: 1115–21. 22371112 10.1007/s00264-011-1459-4 PMC3353094

[14] Nakano N , Lisenda L, Khanduja V et al Complications following arthroscopic surgery of the hip: a systematic review of 36 761 cases. Bone Joint J 2017; 99-B: 1577–83. 29212679 10.1302/0301-620X.99B12.BJJ-2017-0043.R2

[15] Gudipati S , Fragkakis EM, Ciriello V et al A cohort study on the incidence and outcome of pulmonary embolism in trauma and orthopedic patients. BMC Med 2014; 12: 39. 24589368 10.1186/1741-7015-12-39 PMC3996019

[16] Dindo D , Demartines N, Clavien P-A. Classification of surgical complications. Ann Surg 2004; 12: 39. 10.1097/01.sla.0000133083.54934.ae PMC1360123 15273542

[17] Scottish Intercollegiate Guidelines Network. Prevention and Management of Venous Thromboembolism—A National Clinical Guideline. Management; 2008. https://www.sign.ac.uk/our-guidelines/prevention-and-management-of-venous-thromboembolism/. Accessed: 10 January 2020.doi:10.1136/thx.2008.097741.

[18] Scottish Intercollegiate Guidelines Network. *Prevention and Management of Venous Thromboembolism: A National Clinical Guideline.* SIGN, 2010. Available at: https://www.sign.ac.uk/our-guidelines/prevention-and-management-of-venous-thromboembolism/. Accessed: 10 January 2020.

[19] Pollack CV , Schreiber D, Goldhaber SZ et al Clinical characteristics, management, and outcomes of patients diagnosed with acute pulmonary embolism in the emergency department: initial report of EMPEROR (multicenter emergency medicine pulmonary embolism in the real world registry). J Am Coll Cardiol 2011; 57: 700–6. 21292129 10.1016/j.jacc.2010.05.071

[20] Lapidus LJ , Ponzer S, Pettersson H et al Symptomatic venous thromboembolism and mortality in orthopaedic surgery—an observational study of 45 968 consecutive procedures. BMC Musculoskelet Disord 2013; 14: 177. 23734770 10.1186/1471-2474-14-177 PMC3682916

[21] National Clinical Guideline Centre—Acute and Chronic Conditions (UK). Venous Thromboembolism: Reducing the Risk of Venous Thromboembolism (Deep Vein Thrombosis and Pulmonary Embolism) in Patients Admitted to Hospital. London: Royal College of Physicians (UK); 2010. Available at: https://www.nice.org.uk/guidance/cg92. Accessed: 10 January 2020. 23346611

[22] Falck-Ytter Y , Francis CW, Johanson NA et al Prevention of VTE in orthopedic surgery patients. Antithrombotic therapy and prevention of thrombosis, 9th ed: American College of Chest Physicians evidence-based clinical practice guidelines. Chest 2012; 141: e278S–325S. 22315265 10.1378/chest.11-2404 PMC3278063

[23] Rezaie AA , Azboy I, Parvizi J. Venous thromboembolism prophylaxis after hip preservation surgery: a review and presentation of institutional experience. J Hip Preserv Surg 2018; 5: 181–9. 30393544 10.1093/jhps/hny016 PMC6206688

[24] Mohtadi NG , Johnston K, Gaudelli C et al The incidence of proximal deep vein thrombosis after elective hip arthroscopy: a prospective cohort study in low risk patients. J Hip Preserv Surg 2016; 3: 295–303. 29632689 10.1093/jhps/hnw027 PMC5883170

[25] Salvo JP , Troxell CR, Duggan DP. Incidence of venous thromboembolic disease following hip arthroscopy. Orthopedics 2010; 33: 664. 20839705 10.3928/01477447-20100722-10

[26] Alaia MJ , Patel D, Levy A et al The incidence of venous thromboembolism (VTE) after hip arthroscopy. Bull Hosp Jt Dis 2014; 72: 154–8. 25150343

[27] Bolia IK , Fagotti L, Mcnamara S et al A systematic review-meta-analysis of venous thromboembolic events following primary hip arthroscopy for FAI: clinical and epidemiologic considerations. J Hip Preserv Surg 2018; 5: 190–201. 30393545 10.1093/jhps/hny029 PMC6206692

[28] Larson CM , Clohisy JC, Beaulé PE et al Intraoperative and early postoperative complications after hip arthroscopic surgery. Am J Sports Med 2016; 44: 2292–8. 27311412 10.1177/0363546516650885

[29] Khazi ZM , An Q, Duchman KR et al Incidence and risk factors for venous thromboembolism following hip arthroscopy: a population-based study. Arthroscopy 2019; 35: 2380–4.e1. 31395174 10.1016/j.arthro.2019.03.054

[30] Cohen AT , Tapson VF, Bergmann J-F et al Venous thromboembolism risk and prophylaxis in the acute hospital care setting (ENDORSE study): a multinational cross-sectional study. Lancet 2008; 371: 387–94. 18242412 10.1016/S0140-6736(08)60202-0

[31] Tapson VF , Decousus H, Pini M et al Venous thromboembolism prophylaxis in acutely ill hospitalized medical patients. Chest 2007; 132: 936–45. 17573514 10.1378/chest.06-2993

[32] An VVG , Phan K, Levy YD et al Aspirin as thromboprophylaxis in hip and knee arthroplasty: a systematic review and meta-analysis. J Arthroplasty 2016; 31: 2608–16. 27178011 10.1016/j.arth.2016.04.004

[33] Collins JA , Beutel BG, Garofolo G et al Correlation of obesity with patient-reported outcomes and complications after hip arthroscopy. Arthroscopy 2015; 31:57–62. 25218005 10.1016/j.arthro.2014.07.013

[34] Domb BG , Gui C, Hutchinson MR et al Clinical outcomes of hip arthroscopic surgery: a prospective survival analysis of primary and revision surgeries in a large mixed cohort. Am J Sports Med 2016; 44:2505–17. 27590174 10.1177/0363546516663463

[35] Wells PS , Prins MH, Beyer-Westendorf J et al Health-care cost impact of continued anticoagulation with rivaroxaban vs aspirin for prevention of recurrent symptomatic VTE in the EINSTEIN-CHOICE Trial Population. Chest 2018; 154: 1371–8. 30201406 10.1016/j.chest.2018.08.1059

[36] Yeol Lee S , Hyun Ro D, Youb Chung C et al Incidence of deep vein thrombosis after major lower limb orthopedic surgery: analysis of a nationwide claim registry. Yonsei Med J 2015; 56: 139. 25510757 10.3349/ymj.2015.56.1.139 PMC4276747

[37] Douketis JD , Eikelboom JW, Quinlan DJ et al Short-duration prophylaxis against venous thromboembolism after total hip or knee replacement. Arch Intern Med 2002; 162: 1465. 12090882 10.1001/archinte.162.13.1465

[38] Madhusudhan TR , Sinha A, Widdowson D. Deep vein thrombosis in shoulder arthroplasty—a prospective study. BMC Musculoskelet Disord 2013; 14: 139. 23597070 10.1186/1471-2474-14-139 PMC3641960

[39] Scurr JH. How long after surgery does the risk of thromboembolism persist? Acta Chir Scand Suppl 1990; 556: 22–4. 2288177

[40] Planes A , Vochelle N, Darmon JY et al Risk of deep-venous thrombosis after hospital discharge in patients having undergone total hip replacement: double-blind randomised comparison of enoxaparin versus placebo. Lancet 1996; 348: 224–8. 8684199 10.1016/s0140-6736(96)01453-5

[41] Wang A , Hendin A, Millington SJ et al Better with ultrasound. Chest 2020; 157: 574–9. 31634448 10.1016/j.chest.2019.08.2209

[42] Shimabukuro N , Mo M, Hashiyama N et al Clinical course of asymptomatic isolated distal deep vein thrombosis of the leg: a single-institution study. Ann Vasc Dis 2019; 12: 487–92. 31942206 10.3400/avd.oa.19-00128 PMC6957892

[43] Gould MK , Garcia DA, Wren SM et al Prevention of VTE in nonorthopedic surgical patients. Chest 2012; 141: e227S–77S. 22315263 10.1378/chest.11-2297 PMC3278061

[44] Kearon C. Natural history of venous thromboembolism. Circulation 2003; 107: 22I–30. 10.1161/01.CIR.0000078464.82671.78 12814982

[45] Kearon C , Akl EA, Comerota AJ et al Antithrombotic therapy for VTE disease: Antithrombotic therapy and prevention of thrombosis, 9th ed: American College of Chest Physicians evidence-based clinical practice guidelines. Chest 2012; 141: e419S–96S. 22315268 10.1378/chest.11-2301 PMC3278049

[46] Kulshrestha V , Kumar S. DVT prophylaxis after TKA: routine anticoagulation vs risk screening approach—a randomized study. J Arthroplasty 2013; 28: 1868–73. 23796558 10.1016/j.arth.2013.05.025

[47] Radzak KN , Wages JJ, Hall KE et al Rate of transfusions after total knee arthroplasty in patients receiving lovenox or high-dose aspirin. J Arthroplasty 2016; 31: 2447–51. 27554782 10.1016/j.arth.2015.10.023

[48] Schelde AB , Eliasen A, Olesen JB et al Real-world effectiveness and safety of pharmacological thromboprophylaxis in patients undergoing primary total hip and knee arthroplasty: a narrative review. J Orthop 2020; 19: 116–73. 10.1016/j.jor.2019.11.012 PMC6997650 32025127

[49] Truntzer JN , Hoppe DJ, Shapiro LM et al Complication rates for hip arthroscopy are underestimated: a population-based study. Arthroscopy 2017; 33:1194–201. 28259588 10.1016/j.arthro.2017.01.021

