# Supplementary material for: Post-operative oral chemoprophylaxis in patients undergoing hip arthroscopy mitigates VTE risk with a low side-effect profile
Source: J Hip Preserv Surg. 2020 Dec 22;7(3):524–32. doi: 10.1093/jhps/hnaa063 (PMC8081424; doi:10.1093/jhps/hnaa063)
Supplement: hnaa063_Supplementary_Data [file hnaa063_supplementary_data.docx]

**Appendix - Pre-operative VTE form**

**VTE Risk Factor Assessment Sheet**

**File Number**: **Date**:

**Diagnosis:**

**Date of Birth: Gender:** ☐Male ☐Female

**Weight**: kg **Height**: m  **BMI**  kg/m^2^

**Current medication:**

**Risk factors**

*All patients*

☐ Previous history of VTE

☐ Family history of thrombosis

☐ Acute MI

☐ Central venous access

☐ Inflammatory bowel disease

☐ Heart failure/pericarditis

☐ Prolonged immobility/ paralysis

☐ History of malignancy

☐ Nephrotic syndrome

☐ Previous stroke

☐ Hypercoagulable state

☐ Hormone replacement therapy

☐ Anabolic Steroid Use

☐ Other:

*Female patients*

☐ Pregnancy

☐ Oral contraceptive use
